# LocSpeck: A Collaborative and Distributed Positioning System for Asymmetric Nodes Based on UWB Ad-Hoc Network and Wi-Fi Fingerprinting

**DOI:** 10.3390/s20010078

**Published:** 2019-12-21

**Authors:** Mostafa Sakr, Andrea Masiero, Naser El-Sheimy

**Affiliations:** 1 Department of Geomatics Engineering, University of Calgary, 2500 University Drive NW, Calgary, AB T2N 1N4, Canada; elsheimy@ucalgary.ca; 2 Interdepartmental Research Center of Geomatics (CIRGEO), University of Padova, via dell’Università 16, 35020 Legnaro (PD), Italy; masiero@dei.unipd.it

**Keywords:** ultra-wideband (UWB), positioning system, indoor localization, real-time location system, embedded systems, ad-hoc networks

## Abstract

This paper presents LocSpeck, a collaborative and distributed indoor positioning system for dynamic nodes connected using an ad-hoc network, based on inter-node relative range measurements and Wi-Fi fingerprinting. The proposed system operates using peer-to-peer range measurements and does not need ultra-wideband (UWB) fixed anchor, nor it needs a predefined network topology. The nodes could be asymmetric in terms of the available sensors onboard, the computational resources, and the power capacity. This asymmetry adversely affects the positioning performance of the weaker nodes. Collaboration between different nodes is achieved through a distributed estimator without the need of a single centralized computing element. The ranging measurement component of the system is based on the DW1000 UWB transceiver chip from Decawave, which is attached to a set of smartphones equipped with asymmetric sensors. The distributed positioning filter fuses, locally on each node, the relative range measurements, the reading from the internal sensors, and the Wi-Fi received signal strength indicator (RSSI) readings to obtain an estimate of the position of each node. The described system does not depend on fixed UWB anchors and supports online addition and removal of nodes and dynamic node role assignment, either as an anchor or as a rover. The performance of the system is evaluated by real-world test scenarios using a set of four smartphones navigating an indoor environment on foot. The performance is compared to that of a commercial UWB-based system. The results presented in this paper show that weak mobile nodes, in terms of available positioning sensors, can benefit from collaboration with other nearby nodes.

## 1. Introduction

Indoor positioning and localization systems constitute a broad research area that spans different sensing techniques and a multitude of position estimation techniques. The field has been experiencing continuous growth in the last few years, from both the research perspective and equally from the commercial perspective, with the proliferation of positioning and localization systems in indoor environments and the rapid adoption of location-based services (LBS) and real-time location systems (RTLS) in commercial, industrial, emergency response, and military settings [1]. In some situations, the individual nodes may not achieve acceptable positioning accuracy because of the limitations of the available sensors or of the insufficient observations. In operating scenarios, where there is a group of nodes in the same physical proximity, and some of these nodes can position itself with relative accuracy, using relative measurements can augment the stand-alone observations of each node and improve the positioning accuracy of the ensemble [2,3]. The use of relative range measurements introduces an additional constraint to the position estimation filter, which can improve the positioning accuracy of the collaborating nodes [4]. Ultra-wideband (UWB) ranging devices are more immune to multipath errors because it can distinguish between different events with a precise temporal resolution, thanks to its large channel bandwidth. This makes the UWB-based devices capable of achieving centimeter-level ranging accuracy in ideal operating conditions [5].

The objective of this paper is to present LocSpeck, a collaborative and distributed positioning system targeting smartphones and handheld applications, which uses UWB-based relative range measurements, along with Wi-Fi fingerprinting and inertial sensors. The system is evaluated experimentally, and the results are compared to the performance of Pozyx, a commercial UWB-based positioning system. The rest of this section will provide a brief overview of different ranging and positioning techniques used in UWB-based systems, and then it will discuss the different network architectures used for range-based positioning applications.

### 1.1. UWB-Based Ranging

Ultra-wideband positioning and localization systems have been used in different scenarios that require centimeter ranging accuracy with constraints on the cost and the power of the ranging devices. The applications of UWB-based localization systems include first responders in emergency situations, assets tracking and monitoring, medical and wellness applications, security and access control, locating nodes in wireless sensor networks, and for military applications [6,7,8,9].

Ranging using ultra-wideband radios can be performed using different techniques: angle-of-arrival (AOA), received signal strength (RSS), time-difference-of-arrival (TDOA), or time-of-arrival (TOA)/time-of-flight (TOF) [7]. The angle-of-arrival-based systems are complex and require more than one antenna on the same node, increasing the cost and the complexity of the implementation. The time-based approaches are more suited to the UWB systems since the high-bandwidth of the signal can provide very fine spatial resolution in addition to increasing its immunity to multipath effects [10].

The Cramér–Rao bound for the time-of-arrival ranging accuracy using the IEEE 802.15.4a, the predecessor to the IEEE 802.15.4-2011, under single-path additive white Gaussian noise (AWGN) channel model, can be expressed as:

(1) $$\sigma_{R}\geq \frac{c}{2\pi \times \beta \times \sqrt{2\left(SNR\right)}}\;,$$

where $\sigma_{R}$ is the standard deviation of the range estimation R, c is the speed of light, β is the effective bandwidth, and SNR is the signal-to-noise ratio [7,11]. Setting β=500 MHz and SNR=10, the standard deviation of the range estimation $\sigma_{R}=2\;\mathrm{cm}$ [12].

The RSS of the UWB signal is less susceptible to small scale fading compared to narrow-band signals [13] as a result of the large bandwidth of the UWB signals. However, the ranging accuracy achievable using RSS methods decreases with distance [14], making the achievable accuracy less than the accuracy obtained using the time-delay methods. The accuracy of the range measurement using RSS techniques can be expressed as:

(2) $$\sigma_{R}\geq \frac{\ln10}{10}\frac{\sigma_{sh}}{n_{p}}\;d\;,$$

where d is the distance between the two nodes, $n_{p}$ is the path loss factor, and $\sigma_{sh}$ is the standard deviation of the zero-mean Gaussian random variable representing the log-normal channel shadowing effect [7]. This could be sufficient for certain applications that do not require a centimeter ranging accuracy or when the nodes are in close proximity. RSS localization relies on two techniques: range estimation or fingerprinting. The first is range estimation knowing the path-loss-model (PLM) and the channel state information (CSI) [13,15]. The second is signal strength fingerprinting [16], which requires a learning phase to collect the RSS fingerprints along with a set of reference points, then during the localization phase, actual RSS value is compared to the previously generated fingerprint database to estimate the location of the UWB receiver in real-time.

### 1.2. UWB-Based Positioning

Thanks to the high accuracy of UWB time-based range measurements, positioning with a decimeter-level of accuracy is usually achievable by solving a multilateration problem in line-of-sight (LOS) working conditions [17], i.e., when the UWB signal is not obstructed by any obstacle between the anchors and the tracked device. In the typical case of a moving device to be tracked, the positioning problem can be conveniently coped with a proper model of the device dynamic and an Extended Kalman Filter (EKF). The accuracy of the obtained position estimates depends on the geometry of the network nodes [18], and it can be assessed by means of the geometric dilution of precision [19]. Several commercial and research positioning systems, e.g., the Pozyx system, are using a fixed UWB network architecture in order to properly track moving nodes [17].

Since non-line-of-sight (NLOS) measurements are quite frequent in indoor environments, several recent works consider the problem of identifying NLOS measurements [20] or dynamically adapting the measurement variance in the EKF in order to reduce the effect of outliers [21]. Recent feature-based approaches provided encouraging results on the NLOS identification and mitigation by properly analyzing the characteristics of the received UWB signal [19,22]. Machine learning approaches proved to be well suited for identifying NLOS measurements as well, while they currently do not seem to provide significant improvements for NLOS effects mitigation [23,24].

The integration with the information provided by other sensors can be considered to mitigate the UWB measurement error and regularize the estimated trajectory, e.g., pedestrian dead reckoning based on the inertial sensor measurements [25]. Furthermore, a cooperative positioning approach can also be considered when the position of multiple devices has to be simultaneously estimated [26]. Peer-to-peer UWB range measurements can be integrated with inertial sensors to improve the positioning accuracy of a centralized estimator [27].

In contrast to the static network architecture case, this paper considers a network that can dynamically self-adapt to the number of available UWB devices. The main advantage of this approach is that it does not require any a priori knowledge of the number of nodes in the network, hence perfectly adapting to the potentially real scenario where at each time instant users can enter or exit the considered area with tracking devices equipped with UWB transceivers. This work aims at investigating the positioning performance in such case, where the use of a fixed UWB architecture is not required, hence leading to a significant system cost reduction, and the use of a collaborative and approach is expected to support the positioning performance of weak nodes, e.g., nodes provided with fewer sensors/less informative measurements.

### 1.3. UWB Network Architecture

**Figure 1:**
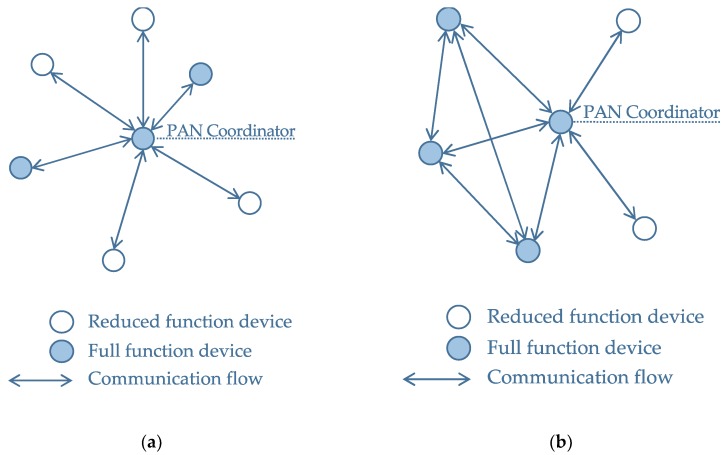
IEEE 802.15.4-2011 supported network topologies [32]: (**a**) Star network topology; (**b**) Peer-to-peer network topology.

The IEEE 802.15.4-2011 standard supports two network architectures for UWB devices in the context of the personal area network (PAN), as shown in Figure 1: the star network architecture, and the peer-to-peer network architecture. A typical network configuration of a UWB-based positioning system comprises a set of fixed anchors and one or more mobile nodes. The position of the anchors is usually known, and the location of the mobile nodes is calculated using the range measured between these nodes and the anchors. An example of a commercial system for positioning, based on the DW1000 [28] UWB radio from Decawave, is the Pozyx system [29,30]. The Pozyx system supports a fixed network architecture [31], such that it requires the prior knowledge of the number of mobile nodes, or tags, and fixed nodes, or anchors, in the network. The position of the tags is calculated sequentially using a time-division-multiple-access (TDMA) approach to eliminate the possibility of interference between the different tags. In the Pozyx system, the positioning and ranging procedures are effectively initiated from a single device in the entire network, which controls the other nodes or tags remotely according to the list of available nodes.

Another aspect of a UWB network architecture is the design of medium access control (MAC) protocols, which has been studied extensively in the context of wireless sensor networks (WSNs). The design of the MAC protocol for sensor networks is guided by the operational goals of the network, and it is usually an attempt to balance between two conflicting goals: achieving high-throughput and maintaining energy-efficient operation. Recent survey articles describe the taxonomy of MAC protocols and outline the development of different MAC protocols [33,34,35,36,37]. The MAC protocols for WSNs can be categorized into synchronous, asynchronous, frame-slotted, and multi-channel protocols. Although the IEEE 802.15.4-2011 standard defines the MAC layer, not every UWB radio chip implements the full standard. For example, the Decawave DW1000 does not implement the MAC layer defined by the standard and leaves this task to the host system [28].

### 1.4. Paper Outline

The rest of this paper describes the architecture of LocSpeck, a distributed and ad-hoc UWB-based positioning system, along with the hardware and software components of the nodes. Section 2 outlines the target network architecture, the hardware architecture of the nodes, and the details of the range measurement message sequence. The ad-hoc medium access protocol of the LocSpeck system is described by the end of Section 2. Section 3 starts by describing the distributed relative-range measurement approach, and then it continues to describe the other measurements used to update the state of the filter, such as the Wi-Fi fingerprints and the floorplan updates. Section 4.1 describes the medium-access protocol simulation environment along with the test setup. This section also discusses the results for the range measurement messages timing and the theoretical limits on the range measurement rate achieved using the proposed medium access protocol. Finally, Section 4.2 describes the collaborative positioning testing environment and the different test scenarios; then, it introduces the positioning results for the proposed system. A brief discussion and the conclusions are presented in Section 5 and Section 6, respectively.

## 2. Ad-Hoc UWB-Based Positioning System

This section describes the hardware and the software aspects of the proposed ad-hoc UWB-based positioning system. The system supports ad-hoc network architecture, run-time inclusion and removal of nodes, and dynamic role assignment to nodes. Furthermore, the proposed system does not depend on fixed anchors nor requires time synchronization between the collaborating nodes. The section starts by describing the supported network structure, then follows by describing the hardware and software underpinnings of the system. The details of the ranging messages and the medium access protocol are discussed later in this section.

### 2.1. Ad-Hoc Network Structure

An ad-hoc network is a network between independent nodes that can change its connectivity dynamically without the need for fixed infrastructure or central control units [38]. The nodes in the LocSpeck system are connected using an ad-hoc network architecture, with no predefined structure nor fixed roles. Since the main objective of the network is to perform ranging measurements between neighboring nodes, within the line-of-sight of the UWB receiver, the ad-hoc network described here has some different characteristics from an ad-hoc network used for communication application [39]. A summary of the network characteristics supported by the proposed positioning system is presented in the following list:

- Flat network topology: the network is composed of symmetric nodes in terms of its communication capability, which means that each node can initiate a ranging request or respond to such requests from other nodes. In addition, there are no coordinating nodes as opposed to the peer-to-peer network architecture described in the IEEE 802.15.4-2011 standard [32,40]. However, the sensing and computational capabilities of the nodes can still be asymmetric.
- Single-hop network: the nodes are only interested in exchanging ranging messages with their neighboring nodes.
- Energy conservation: after either a failed or a successful ranging exchange attempt, the radio chip goes to sleep for a predefined period of time before it can engage in a new ranging sequence.
- Flexibility: nodes can enter and exit the network in real-time, with no need to reconfigure or notify the existing nodes.

**Figure 2:**
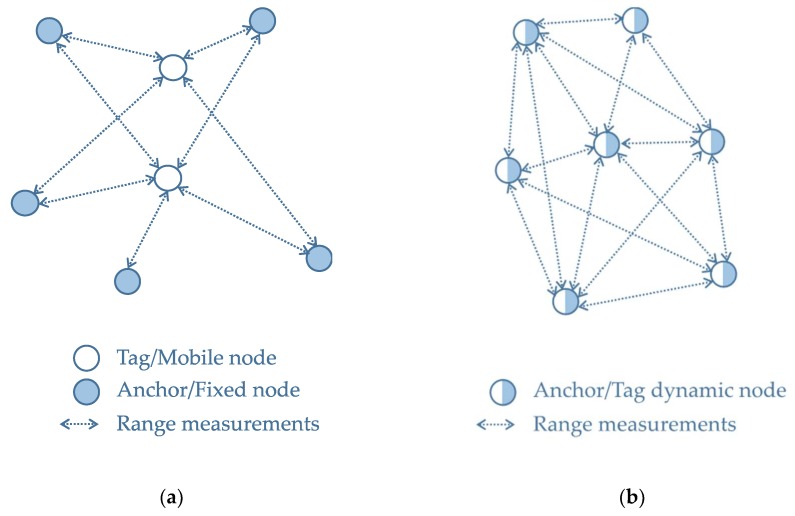
Ultra-wideband (UWB)-based network architecture for ranging and positioning applications: (**a**) Fixed role network; (**b**) Dynamic role network.

These characteristics emphasize the main objective of the network: ranging and positioning. The nodes are identical, in terms of communication capabilities, and could be spread over a large physical space. The differences between the ad-hoc network topology and the fixed-role network topology for ranging and positioning applications can be seen in Figure 2.

### 2.2. Dynamic Nodes Architecture

**Figure 3:**
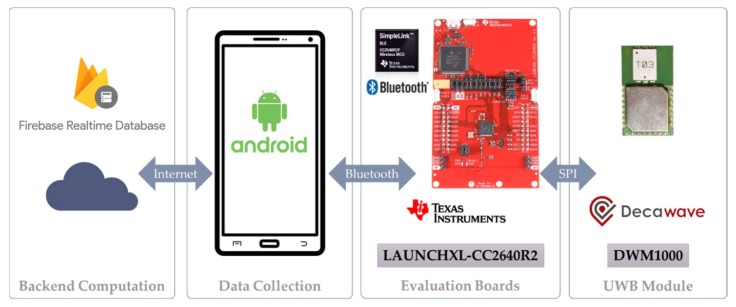
LocSpeck platform overview [41,42].

**Figure 4:**
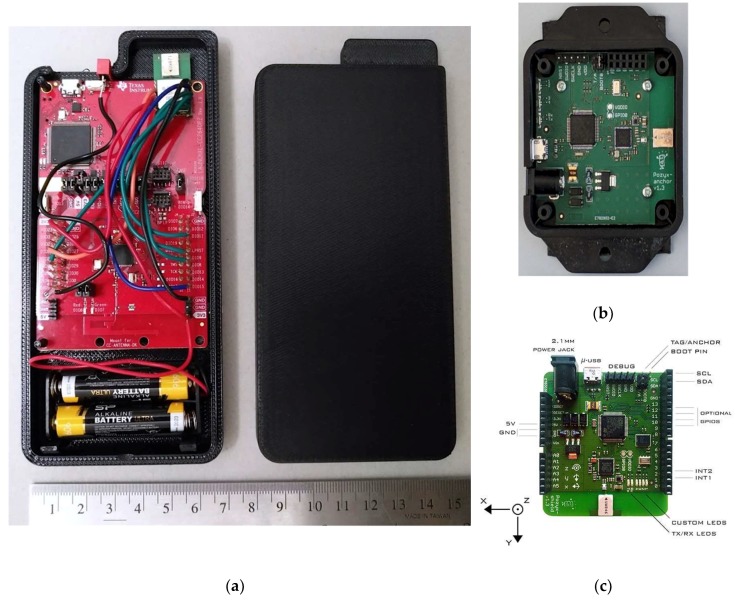
The LocSpeck ranging devices: (**a**) Ranging devices for the proposed UWB-based positioning system; (**b**) Pozyx positioning system–anchor node; (**c**) Pozyx positioning system–tag node [29].

The dynamic nodes are the building blocks of the proposed ad-hoc UWB positioning system. Each node is composed of the ranging device and an associated smartphone. An overview of the architecture of the system is shown in Figure 3. The ranging devices are based on commercial off-the-shelf components. The UWB radio module used is the DWM1000, which is based on the Decawave DW1000 radio and equipped with an on-board chip antenna [41]. The DWM1000 is attached to a CC2640R2 LaunchPad kit from TI [42], which hosts a CC2640R2F wireless microcontroller unit (MCU), enabling a smartphone to communicate with the DW1000 device over a Bluetooth low-energy (BLE) interface. The UWB module and the MCU are connected through a serial peripheral interface (SPI), allowing the host MCU to configure the UWB radio, initiate range measurements, and obtain data and status information from the radio. The UWB module and the BLE evaluation board from TI are enclosed in a custom-built plastic case, which hosts the batteries and the power switch, as shown in Figure 4a. The components of the systems are selected to ensure centimeter ranging accuracy, provided by the Decawave radio module. The BLE interface is used to easily pair a UWB module to a smartphone, which provides a low-cost and versatile platform with sensing, computing, and communication capabilities. For comparison, the anchor and the tag nodes of the Pozyx system are shown in Figure 4b,c.

**Figure 5:**
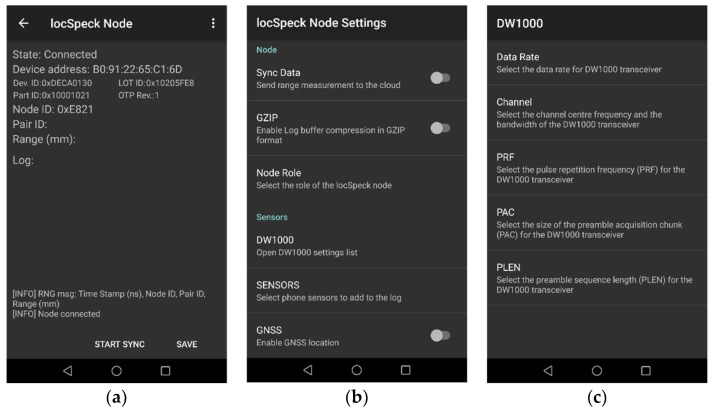
The LocSpeck logging Android application: (**a**) Data logging screen; (**b**) LocSpeck node settings screen; (**c**) Decawave DW1000 settings screen.

The host smartphone connects to the ranging device through a Bluetooth Low Energy (BLE) interface—the smartphone also runs the sensor logging application, as shown in Figure 5. The ranging device firmware and the logging application communicates through the Generic Attribute Profile (GATT), which is a component of the Bluetooth low-energy protocol stack responsible for the actual data exchange between any two connected devices. The GATT stores and passes the data as a set of fields, called characteristics, which are stored in the memory of the BLE device. The GATT profile implemented by the LocSpeck ranging device is summarized in Table 1. The LocSpeck logging application can identify the attached UWB chip, read and change the configuration of the UWB chip, and log the range and the paired node ID. The LocSpeck logging application can collect data from the smartphone sensors (e.g., accelerometer, gyroscope, magnetometer, Wi-Fi RSSI, barometers), and it can record the GNSS position information when it is available. The LocSpeck logging software is capable of synchronizing the local measurements using a cloud-based real-time database. This capability can be used to enable a centralized approach for nodes positioning. However, for the rest of this work, the positioning filters are implemented in a distributed fashion on each node. The collaboration between different nodes is achieved through the exchange of the relative range measurements between the collaborating nodes.

### 2.3. Range Measurement Messages

The ability of UWB systems to provide accurate ranging is the motivation to use them for indoor positioning and localization. The relative range measurements between two UWB transceivers can be achieved using delay or time-based methods, angle-of-arrival methods, or received signal strength methods [7]. As shown earlier in Equations (1) and (2), the Cramér–Rao lower bound for range measurements using the received signal strength increases with the distance, lowering the achievable positioning accuracy. The angle-of-arrival method requires antenna arrays to distinguish the phase of each incident radio rays to calculate the relative angle between nodes. The superior theoretical performance and the simpler implementation renders the time-based ranging solution an attractive option for commercial positioning solutions such as receivers from Decawave, BeSpoon, and Ubisense [17], though the Ubisense system supports the angle-of-arrival measurements as well.

Before proceeding to describe the range measurement algorithm implemented by the LocSpeck node, two time-based ranging methods will be briefly discussed: the time-difference-of-arrival (TDOA) and the time-of-flight (TOF) methods. In TDOA-based systems, the tag or the mobile node sends a periodic message which is received by the surrounding anchors. The internal clocks of the anchors must be synchronized, so the anchors can compare the arrival time of the tag message using the same time reference [43], and find the position of the tag. In TOF-based systems, two-way communication between the neighboring nodes is required to calculate the time-of-flight without the need for synchronizing the clocks of different nodes. The time-of-flight is converted to a range measurement by multiplying it by the speed of light. The DW1000 chip can implement both methods. However, the LocSpeck system implements the time-of-flight method for range calculation.

**Figure 6:**
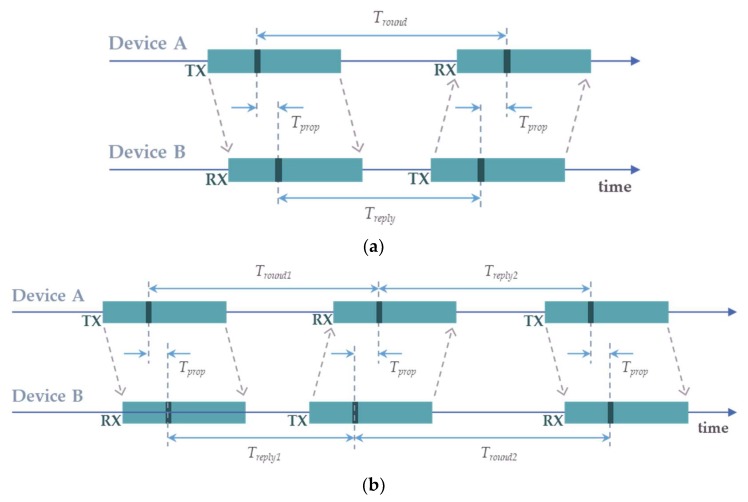
Two-way ranging frame sequence [28]: (**a**) Single-sided two-way ranging; (**b**) Asymmetric double-sided two-way ranging.

Figure 6 shows the message exchange sequence for two different time-of-flight ranging techniques utilizing the Decawave DW1000 chip. Although the DW1000 chip does not implement the top-level ranging technique, the chip provides means to precisely control the messages exchange and to accurately time-stamp each transaction [28]. The host system—in the case of LocSpeck, the ARM Cortex-M3 MCU embedded on the TI CC2640R2 chip—is responsible for implementing the range measurement algorithm. Figure 6a shows the messages exchanged between two nodes for the single-sided two-way ranging. The propagation time can be calculated using Equation (3):

(3) $$T_{prop}=\frac{1}{2}\left(T_{round}-T_{reply}\right)\;,$$

where $T_{round}$ and $T_{reply}$ are the round-trip time and reply time, respectively. Each time quantity is measured on device A and device B using their local clocks, alleviating the need to synchronize the nodes. This method represents a simple approach to calculating the range, with the exchange of two messages only. However, the drawback of the single-sided ranging method is that the error in the range measurements increases as the reply time increases. The reason for this error is attributed to the small clock offset from its nominal value in the oscillator of each chip.

Figure 6b shows the asymmetric double-sided two-way ranging method in which the ranging exchange requires three messages, where two round trips are combined to calculate the propagation time, reducing the ranging errors [28]. The asymmetry in this exchange is manifested by the fact that the reply time of both nodes is not equal. The propagation time for the asymmetric two-way ranging can be calculated using Equation (4):

(4) $$T_{prop}=\frac{T_{round1}\times T_{round2}-T_{reply1}\times T_{reply2}}{T_{round1}+T_{round2}+T_{reply1}+T_{reply2}}\;.$$

**Figure 7:**
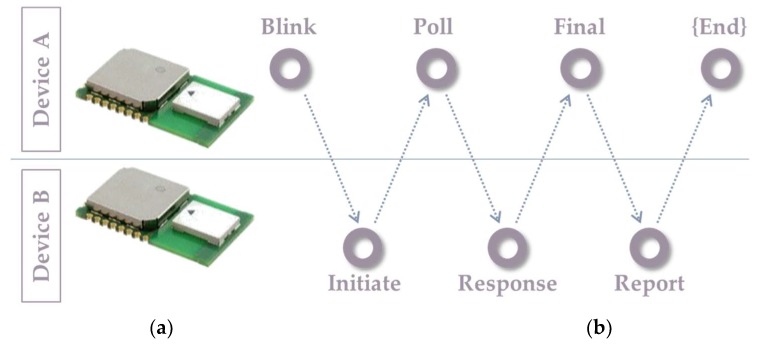
Ranging message structure: (**a**) Decawave DWM1000 modules, based on DW1000 UWB radio chip; (**b**) Ranging messages exchanged between two nodes.

The propagation time calculated in Equation (4) ensures that the error due to clock offset is minimized compared to the single-sided method. The LocSpeck node implements the double-sided two-way method using three messages for ranging in addition to one final message to share the calculated range between the nodes pair, as shown in Figure 7. In this example, the messages exchange is expressed as follows:

1. Device A begins the ranging exchange by sending a blink message to any of the surrounding nodes. The purpose of this message is to notify any available nodes that device A is prepared to proceed with the range measurement exchange.
2. If device B is within the communication range and is listening to the correct UWB channel, it receives the blink frame and replies by sending the range measurement initiation message, using the address of device A.
3. Device A receives the ranging initiation message, then it sends back a poll message to the other side and records the precise time of sending the poll frame.
4. Device B gets the poll message and stamps the arrival time. Then, device B sends a response message to device A and record the reply time ($T_{reply1}$).
5. Device A gets the response frame and saves the arrival time stamp, and then calculates the first round-trip time ($T_{round1}$). After the second reply time ($T_{reply2}$), device A sends the final message.
6. Device B receives the final frame and records the round-trip time ($T_{round2}$). Using Equation (4), device B calculates the propagation time ($T_{prop}$), and consequently, the range.
7. Finally, device B sends the propagation time back to device A.

By the end of the messages exchange, the measured range value is available at both devices for further processing. The positioning algorithm uses the range information along with the other local sensors readings to update the position state of the nodes.

### 2.4. Medium Access Protocol

**Figure 8:**
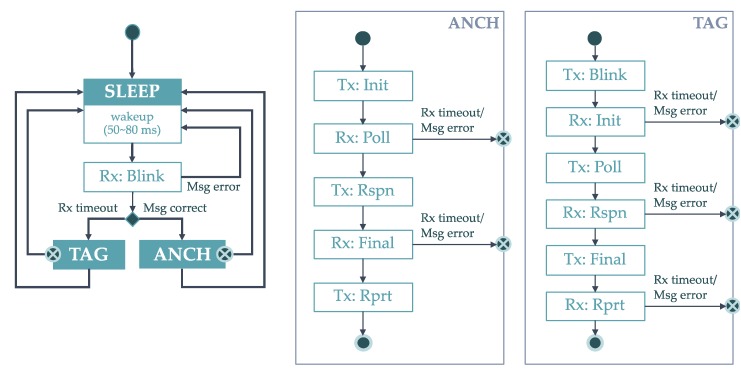
LocSpeck medium access protocol.

The LocSpeck nodes are designed to operate in an unpredictable environment, in terms of the number of the surrounding nodes and in terms of the possible structure of the network formed using these nodes. The nodes should support rapid deployment with no or minimum effort from the operator. To meet the target operating conditions, LocSpeck nodes implement a simple and light-weight random-access medium access protocol based on the pure ALOHA protocol [44,45]. Figure 8 shows an overview of the medium access protocol implemented by the LocSpeck nodes.

After powering up LocSpeck nodes, they start in the sleep state. Each node sleeps for a random duration between 50 ms and 80 ms. When the sleep duration elapses, nodes wake-up and power-up its receiver, waiting for any incoming frames. If a node receives a complete frame, it decodes the frame and checks if the incoming frame is a blink message. In the case of receiving a blink message, the node operates as an anchor. If the node receives a complete frame, and it is not a blink message, it concludes that the channel is currently occupied with the ranging sequence of another pair of nodes, and goes back to the sleep state, so it would not interfere with the current exchange. If no frames are received and the listening period elapses, the receiver timeout flag is asserted. In this case, the node assumes that the channel is free, so it switches to the tag mode and starts sending a blink message to any active node.

While at the anchor or the tag states, each node sends or receives a sequence of messages. If any of the received messages does not match the expected message at that stage, the node will switch back to sleep mode. Also, the nodes will go to sleep mode, if there is any problem with messages transmitting or receiving, such as a receiver timeout, or any other problem related to the radio interface. Once a ranging sequence is completed successfully, the node saves the range and the node ID of the collaborator. Finally, each node sends a notification to the host smartphone before returning to the sleep mode for another random duration.

The LocSpeck medium access protocol assumes that all the nodes use the same UWB channel and the same preamble code for both communications and for ranging. The DW1000 supports the use of 6 RF channels out of the 16 channels defined in the IEEE 802.15.4-2011 standard. Each node operating in the channel is assigned a preamble code from a set of two or four possible codes. The exact values of the preamble sequence are defined by the standard and are selected to ensure minimum cross-correlation between different codes. Assigning different nodes to different channels and assigning different preamble codes to the nodes operating in the same channel can increase the number of nodes operating in close proximity [46]. Although the multiple-channel and multiple-preamble approach can increase the effective number of nodes, its use was not considered in this work since it would increase the complexity of the system. Using this approach will require more functionality on top of the current protocol to scan different channels and preambles and to keep track of nodes in each channel-preamble configuration.

## 3. Collaborative Positioning Algorithm

This section describes the two-dimensional positioning algorithm implemented in the LocSpeck system. It highlights the dynamic motion model of the nodes, along with the measurements update model. The LocSpeck nodes use different measurement update models: relative range measurement updates, Wi-Fi fingerprinting updates, and map information update.

The standalone position algorithm is implemented using a particle filter (PF) which performs better than the extended Kalman filter (EKF) or the unscented Kalman filter (UKF), due to the non-linear nature of the Wi-Fi fingerprinting update [47]. The PF version used for this work uses a pedestrian dead-reckoning (PDR) algorithm for the state update using input from the gyroscope and the accelerometer if these sensors were available on the host smartphone. The filter uses Wi-Fi fingerprinting to update the weights of the particles, using a Gaussian process model as the reference map. The implementation details of the standalone positioning filter, including the pedestrian dead-reckoning and the Gaussian process-based fingerprinting, were discussed in [48]. The standalone filter runs on each node independently, where the processing of the relative range measurement and the local sensors measurements are handled on each node.

The weights of the particles in the filter are updated using the relative-range, the Wi-Fi received signal strength indicator (RSSI) measurements, and the map information, using Equation (5):

(5) $${\tilde{w}}_{k}^{i}\propto w_{k-1}^{i}\times p\left(z_{k}^{R}|x_{k}^{i}\right)\;,$$

where $w_{k-1}^{i}$ is the current weight of particle i, ${\tilde{w}}_{k}^{i}$ is the updated weight, $z_{k}^{R}$ is the value of the observation, and *p* $\left(z_{k}^{R}|x_{k}^{i}\right)$ is the likelihood of the observation $z_{k}^{R}$ at the location defined by the horizontal coordinates of the particle i, $x_{k}^{i}$.

Equation (5) is a simplified version of the particle filter weights update equation in which the proposal density is the state transition model. After the weight update step, a weight normalization and resampling steps are implemented to remove the undesired particles. The filter is implemented with relatively low particle count (150 particles) to reduce the processing time required. The details of the measurement likelihood equations for different measurement updates equations are presented in the following subsections.

### 3.1. Distributed Relative-Range Measurement Update

The measurement update using a relative-range between a pair of collaborating nodes involves two pieces of information: the relative-range and the position of the collaborating node. The measurement can be described using Equation (6).

(6) $$h_{k}^{R,i}\left(x_{k}^{i},x_{k}^{c}\right)={\left\|x_{k}^{i}-x_{k}^{c}\right\|}_{2}\;,$$

where $h_{k}^{R}\left(\cdot \right)$ is the range measurement estimate at time step *k*; $x_{k}^{i}$, is the position of the particle i; and $x_{k}^{c}$ is the coordinates of the collaborating node. The range estimate is used to evaluate the likelihood of the range measured given the position of each particle, $p(z_{k}^{R}|x_{k}^{i},x_{k}^{c})\;$, as described by Equation (7).

(7) $$p\left(z_{k}^{R}|x_{k}^{i},x_{k}^{c}\right)={\left(2{\pi \sigma }_{R}^{2}\right)}^{-\frac{1}{2}}\exp\left(-\frac{1}{2}\frac{{\left(z_{k}^{R}-h_{k}^{R,i}\right)}^{2}}{\sigma_{R}^{2}}\right)\;,$$

where $z_{k}^{R}$ is the actual range measurement, which is modeled as a Gaussian random variable with mean equals to the measured range and covariance of $\sigma_{R}^{2}$. 

Note that Equation (7) not only depends on the local node state, but it also depends on the collaborating node state. In this case, the particle filter running locally on each node needs to account for the collaborating node uncertainty and the cross-correlation that stems from the collaboration between different nodes.

This is accomplished by considering the joint distribution of the state of the local and the collaborating nodes, conditioned on the relative range measurement, $p\left(x_{1:k},x_{k}^{c}|z_{1:k}^{R}\right).$ The problem is further simplified by utilizing the Rao–Blackwellized particle filter (RBPF) formulation [49,50], in which the joint distribution can be factored into a conditionally linear component and a nonlinear component:

(8) $$p\left(x_{1:k},x_{k}^{c}|z_{1:k}^{R}\right)=p\left(x_{k}^{c}|x_{1:k},z_{1:k}^{R}\right)p\left(x_{1:k}|z_{1:k}^{R}\right),$$

where the local state, $x_{1:k}$ is the nonlinear component, and its probability distribution is represented by the particles. The state of the collaborating node, $x_{k}^{c}$, is the conditionally linear component.

Following the RBPF formulation, the marginalized particle filter equation, $p\left(x_{1:k}|z_{1:k}^{R}\right)$, can be represented in Equation (9).

(9) $$p\left(x_{1:k}|z_{1:k}^{R}\right)\propto p\left(x_{k}|x_{k-1}\right)p\left(x_{1:k-1}|z_{1:k-1}^{R}\right)\int p\left(z_{k}^{R}|x_{k},x_{k}^{c}\right)p\left(x_{k}^{c}|x_{1:k},z_{1:k-1}^{R}\right)dx_{k}^{c}\;,$$

where $p\left(x_{1:k}|z_{1:k}^{R}\right)$ is the marginalized posterior of the local state conditioned on the relative range measurement.

As shown in Equation (9), the local particle filter keeps track of the local state only, and the state of the collaborating nodes need to be sent over the communication channel after each collaboration exchange. Sending the full posterior, represented as particles, will consume the available bandwidth by occupying the radio channel for more time. Instead, each node stores a simplified representation of its posterior as a Gaussian distribution, which can be fully described by the mean and variance of the node position. The mean of the state can be described by Equation (10).

(10) $${\hat{x}}_{k}=\overset{N}{\underset{i=1}{\sum }}w_{k}^{i}x_{k}^{i}\;,$$

where $w_{k}^{i}$ is the weights of the particles, and N is the number of particles.

The variance of the state is described by Equation (11).

(11) $$cov\left(x_{k},x_{k}\right)=\left(\overset{N}{\underset{i=1}{\sum }}w_{k}^{i}x_{k}^{i}{\left(x_{k}^{i}\right)}^{T}\right)-{\hat{x}}_{k}{\left({\hat{x}}_{k}\right)}^{T}\;,$$

Similarly, the cross-covariance matrix expression is given by Equation (12).

(12) $$cov\left(x_{k},x_{k}^{c}\right)=\left(\overset{N}{\underset{i=1}{\sum }}w_{k}^{i}x_{k}^{i}{\left(x_{k}^{c,i}\right)}^{T}\right)-{\hat{x}}_{k}{\left({\hat{x}}_{k}^{c}\right)}^{T},$$

The values represented by Equations (10) and (11) are exchanged between the different nodes, along with the measured relative range, $z_{k}^{R}$. When collaboration is initiated between different nodes, each node has to keep track of the collaborating nodes and update the cross-covariance, Equation (12), between the local state and the state of the collaborating nodes, $cov\left(x_{k},x_{k}^{c}\right)$. This approach is inspired by the Schmidt–Kalman filter approach [51] but extended to fit a particle filter framework.

When the local node initiates a new range measurement with another node, the conditional distribution, $p\left(x_{k}^{c}|x_{k}^{i}\right)$, in Equation (9), can be expressed as a Gaussian distribution, $N\left(x_{k}^{c};{\bar{x}}_{k}^{c,i},{\bar{P}}_{k}^{c}\right)$, were ${\bar{x}}_{k}^{c,i}$ and ${\bar{P}}_{k}^{c}$ are evaluated using Equations (13) and (14), respectively [52].

(13) $${\bar{x}}_{k}^{c,i}=x_{k}^{c}+cov\left(x_{k-1},x_{k-1}^{c}\right){\left(cov\left(x_{k-1},x_{k-1}\right)\right)}^{-1}\left(x_{k}^{i}-{\hat{x}}_{k-1}\right)\;,$$

(14) $$\;{\bar{P}}_{k}^{c}=P_{k}^{c}-cov\left(x_{k-1},x_{k-1}^{c}\right){\left(cov\left(x_{k-1},x_{k-1}\right)\right)}^{-1}{\left(cov\left(x_{k-1},x_{k-1}^{c}\right)\right)}^{T}\;,\;$$

Algorithm 1 summarizes the steps to perform the distributed relative-range measurements update, taking into consideration the uncertainty in the collaborating node and the possible cross-correlation between the two nodes:
**Algorithm 1** Distributed Relative-Range Measurement Update**Input:**Range measurement and collaborating node state parametrized with the mean and the covariance: $\left\{z_{k}^{R},{\hat{x}}_{k}^{c},\;cov\left(x_{k}^{c},x_{k}^{c}\right)\right\}\;$
**Output:**Local state posterior, $p\left(x_{k+1}|x_{k},z_{k}^{R}\right)\;$, and cross-covariance, $cov\left(x_{k+1},x_{k+1}^{c}\right)\;$
1 **for** each particle i 
2   Evaluate the conditional distribution, $p\left(x_{k}^{c}|x_{k}^{i}\right)\;$, Equations (13) and (14)3   Evaluate the measurement likelihood, $p(z_{k}^{R}|x_{k}^{i},x_{k}^{c})\;$, Equation (7)4   Evaluate the marginal likelihood: $p\left(z_{k}^{R}|x_{k}^{i}\right)=\int p\left(z_{k}^{R}|x_{k}^{i},x_{k}^{c}\right)p\left(x_{k}^{c}|x_{1:k}^{i},z_{1:k-1}^{R}\right)dx_{k}^{c}\;$
5   Evaluate $p\left(x_{k}^{c,i}|x_{1:k}^{i},z_{1:k}^{R}\right)\;$, using RBPF formulation [49,50]6   Evaluate particle weight: ${\tilde{w}}_{k}^{i}\propto w_{k-1}^{i}\times p\left(z_{k}^{R}|x_{k}^{i}\right)\;$
7   Time update step: $p\left(x_{k+1}^{i}|x_{k}^{i},z_{k}^{R}\right)$ and $p\left(x_{k+1}^{c,i}|x_{1:k}^{i},z_{1:k}^{R}\right)\;$
8 **end for**9 Normalize the particle weights: $w_{k}^{i}={\tilde{w}}_{k}^{i}/\underset{i}{\sum }{\tilde{w}}_{k}^{i}\;$
10  Evaluate the mean and variance terms, ${\hat{x}}_{k+1}$ and $cov\left(x_{k+1},x_{k+1}\right)\;$, Equations (10) and (11).11  Update state cross-covariance term: $cov\left(x_{k+1},x_{k+1}^{c}\right)\;$, Equation (12)12  **return**
$p\left(x_{k+1}|x_{k},z_{k}^{R}\right)$ and $cov\left(x_{k+1},x_{k+1}^{c}\right)\;$


The previous discussion illustrates that the proposed positioning system is a distributed system, which does not need a centralized processing element to estimate the state of the collaborating nodes. Each local node keeps track of the collaborating nodes and keeps track of the cross-covariance between its local state and the state of the collaborating nodes.

### 3.2. Wi-Fi RSSI Fingerprint Update

The Wi-Fi RSSI fingerprinting method used in this work employs a Gaussian process model to represent the RSSI map [48]. The Gaussian process model is a non-parametric model, which is fully defined, in terms of a set of training data, by a mean function and a covariance function, $\mu_{wifi}$ and $\sigma_{wifi}^{2}$ respectively [53]. Using the mean and covariance functions, the likelihood of observing a certain set of Wi-Fi RSSI values can be described using Equation (15):

(15) $$p\left(z_{k}^{wifi}|x_{k}^{i}\right)=\det{\left(2\pi \Sigma_{wifi}\right)}^{-\frac{1}{2}}\;\exp\left(-\frac{1}{2}{\left(z_{k}^{wifi}-M_{wifi}\right)}^{T}\Sigma_{wifi}^{-1}\left(z_{k}^{wifi}-M_{wifi}\right)\right)\;,$$

where $z_{k}^{wifi}$ is a vector with all the observed RSSI values at time step k, $M_{wifi}$ is a vector of the mean function values for each observed Wi-Fi access point (AP), i.e., $M_{wifi}\;=\;\left[\mu_{wifi}^{1},\ldots ,\mu_{wifi}^{N}\right]$, and $\Sigma_{wifi}$ is the observations covariance matrix, which is a diagonal matrix with each element in the diagonal represents the covariance value for each observed AP, i.e., $\Sigma_{wifi}\;=\;\mathrm{diag}\left(\left[{\left(\sigma_{wifi}^{1}\right)}^{2},\ldots ,{\left(\sigma_{wifi}^{N}\right)}^{2}\right]\right)$. The mean and variance functions for each access point, $M_{wifi}$ and $\Sigma_{wifi}\;$, are constructed using a set of training data, $D\;=\;\left\{\left(x_{1},z_{1}\right),\left(x_{2},z_{2}\right),\ldots ,\left(x_{N},z_{N}\right)\right\}$, where, $x_{n}$ is the horizontal position of the training data, i.e., $x_{n}\in R^{2}$, and $z_{n}$ is the RSSI value vector, measured at the point $x_{n}$. More details about the implementation of the Wi-Fi RSSI model using the Gaussian process model can be found in [48].

### 3.3. Floorplan Update

The filter also uses floorplan information to ensure that the effective particles are contained within the area of interest and to eliminate the out-of-bound particles. Equation (16) shows the weight update equation using the floorplan information:

(16) $${\tilde{w}}_{i}=\{\begin{array}{c} \begin{array}{cc} w_{i}, & x_{i}\in FP \end{array}\\ \begin{array}{cc} 0, & x_{i}\notin FP \end{array} \end{array}\;,$$

where $x_{i}$ is the position of the i-th particle, with weight $w_{i}$, and FP is the floorplan.

## 4. Experiments and Results

This section summarizes the performance results for several aspects of the LocSpeck positioning system. It highlights the performance of the different components of the system: the timing of the ranging frames, the efficiency of the medium access protocol, and finally, the positioning accuracy of the LocSpeck system using several realistic test scenarios.

### 4.1. Medium Access Protocol Performance

#### 4.1.1. Range Measurement Messages Timing

The timing characteristics of the ranging sequences and the individual components of the sequence were measured and analyzed using DW1000 on-chip high precision clock for time-stamping the different sequences. To obtain the range and ranging frames duration, two LocSpeck devices were placed 60 cm apart, and the firmware was modified to enable logging the frame duration measurements to a computer. The total duration of the ranging frame is affected by the DW1000 chip settings, such as the preamble length and the data rate. The DW1000 chip settings used for the rest of this section are summarized in Table 2. Changing the PRF, PLEN, and DR not only affects the frame time, but it can also affect the ranging performance.

**Figure 9:**
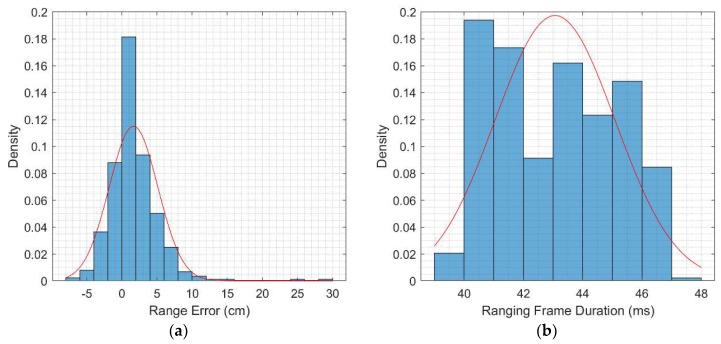
Range measurement frame statistics: (**a**) Range error probability density function (pdf), (**b**) Ranging frame duration pdf.

Figure 9 shows the histograms of the error of the range measurement values and the total duration of the ranging frames for a sample of approximately 430 range measurements. The size of the data payload of each message in the ranging sequence and the duration of each message is listed in Table 3. These values were calculated according to the active DW1000 chip settings and the size of the payload data [54]. The measured ranges using the DW1000 can be affected by noise, uncalibrated bias, and received signal power-dependent biases [55]. The overall ranging frame duration is dominated by the messages sending time and by the processing delays mandated by the firmware implementation and the processor speed. Since the host microcontroller is handling multiple tasks concurrently, the total ranging frame time will account for any other background tasks running during the ranging sequence. The propagation time of the messages from one node to another is 2 ns, which is negligible relative to the transmission and the processing delays.

Figure 9 shows the distribution of both the range error and the ranging frame duration. With 60 cm separation between the two nodes, the mean of the measured range error is 1.6 cm, with a 3.5 cm standard deviation. This error can be attributed to multiple factors: residual biases after node range calibration and measurement setup inaccuracies. The residual bias arises from the fact that the bias is modeled as a constant value, whereas the bias is dependent on the power level of the received signal or equivalently on the separation between nodes. The mean and variance of the range error and frame duration are summarized in Table 4.

**Figure 10:**
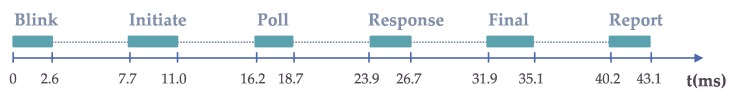
Messages exchange timeline of the ranging frame.

Figure 10 shows the sequence of the messages exchanged during the ranging frame along the time axis. This frame structure is used in evaluating the LocSpeck medium access protocol, as discussed in Section 4.1.2. The messages duration was calculated, as highlighted in Table 3, while the average processing time between messages was calculated using the total ranging frame measurements, captured using the DW1000 precise timing capabilities, and distributed equally among the processing gaps between different messages transmission.

#### 4.1.2. Medium Access Protocol Performance

This section examines the performance of the LocSpeck medium access protocol in terms of the ranging efficiency as a function of the number of the collaborating nodes. It is expected in a random-access protocol such as ALOHA, the basis of the LocSpeck medium access protocol, that the utilization of the medium is reduced due to collisions between different nodes attempting to initiate ranging sequence at the same time. Using the settings outlined in Table 2, the theoretical maximum ranging rate achievable by the LocSpeck system is 20 measurements per second. The ranging rate is calculated using the average frame duration in addition to 15% of the ranging frame used as a guard interval between different ranging frames, as shown in Table 5.

**Figure 11:**
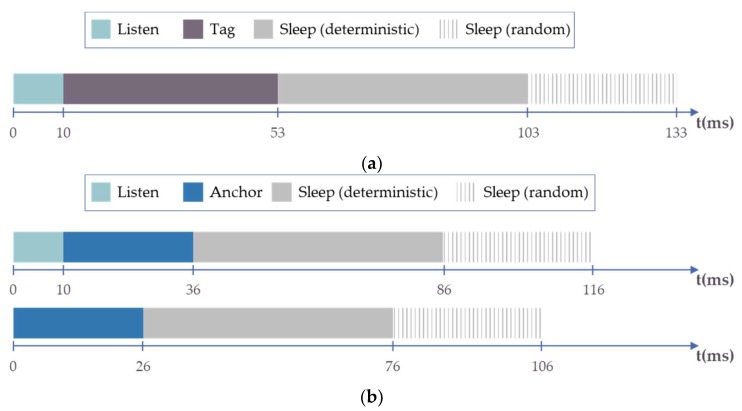
Dynamic node role transition: (**a**) Tag node role, (**b**) Anchor node role.

Figure 11 shows the role transition of nodes with time according to the LocSpeck medium access protocol, as outlined in Figure 8. Figure 11a shows the state transition for a node acting as a tag. At time 0, the node wakes up and starts listening for any incoming messages. If no messages were received after 10 ms, the node concludes that the channel is free and ready for a new transmission. The node role switches to tag and starts the ranging sequence by sending a blink message. If there is a listening node in the tag proximity, it sends a response, and the ranging sequence will continue for another 43 ms. After finishing the ranging sequence, the node sleeps for 50 to 80 ms. The sleep interval is a random value that changes every time a node enters sleep mode. Since the frame duration and the sleep interval is between 103 and 133 ms, the theoretical ranging rate between two LocSpeck nodes is 7.5 to 9.7 measurement per seconds. The maximum ranging rate over the channel with the LocSpeck medium access protocol is 18.9 measurement per second, assuming a new range measurement will start once the active node goes to sleep mode, which occurs every 53 ms. Figure 11b shows the timeline when a LocSpeck node switches to the anchor role. This switch will occur if the node detects a valid blink message during the listen interval. Since the blink message can be received at any point during the listen interval, the complete frame duration for the node in anchor role ranges between 76 to 116 ms, considering the uncertainty in the first 10 ms listening interval and the uncertainty in the 30 ms sleep random component.

**Figure 12:**
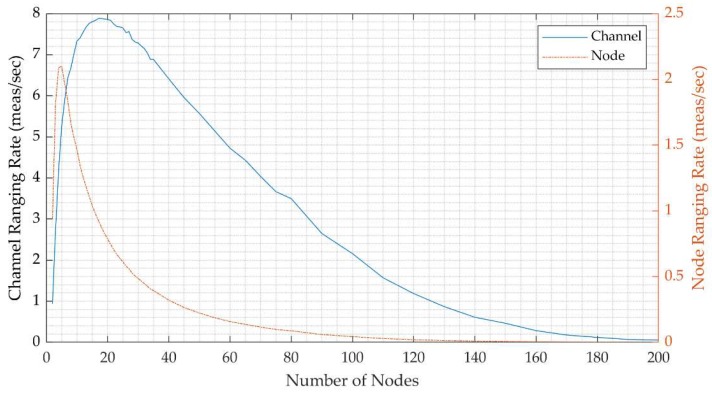
Simulated ranging rates results: the average ranging rate over the channel for all nodes and the average ranging rate per node.

Figure 12 shows the simulated ranging rates versus the number of collaborating nodes, including the channel ranging and the node ranging rates. The maxima of the raging rates are summarized in Table 6. The channel ranging rates as a function of the number of nodes is shown as a solid line in Figure 12. Under the tested configuration of the system, the maximum utilization of the channel is achieved with 18 nodes, reaching 7.9 measurements per second, which accounts for channel efficiency of 39.5% compared to the theoretical 20 measurements per second. It is worth noting that given fixed time, the ranging rate drops with the number of nodes until it is practically zero. The dotted line in Figure 12 shows the average ranging rate per node, assuming range measurements are uniformly distributed across the node population. The maximum ranging rate per node is 2.1 measurements per second, with five active nodes achieving 10.4% of the theoretical rate.

### 4.2. Positioning and Localization Performance

**Figure 13:**
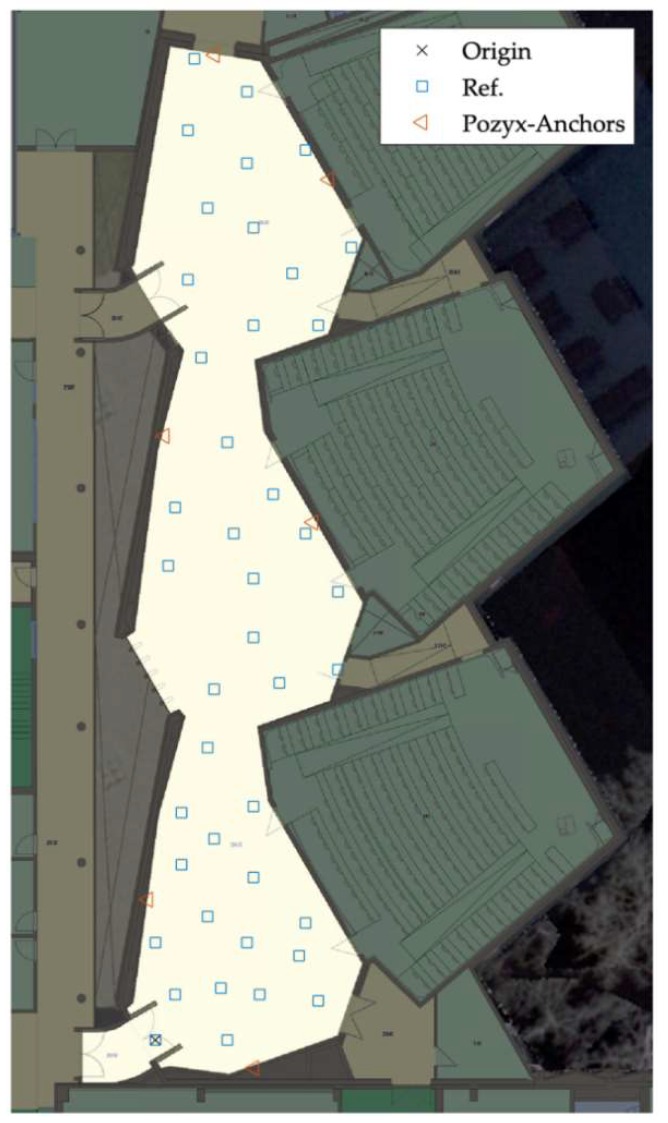
Testing environment floorplan.

This section describes the positioning results of the LocSpeck system and compares the results with those obtained by the Pozyx system. The experiment took place on the second floor of the engineering block E (ENE) building at the University of Calgary. The surface area of the testing region is 360 m^2^, the length of the testing region is 48 m, and the average width is 7.5 m. Figure 13 shows the floorplan of the testing area, the locations of the Pozyx fixed anchors, and the locations of the reference points fixed on the floor of the testing area.

The experiments were carried out using four dynamic nodes—one of them is the main node, while the other three nodes are the supporting nodes. Each node consists of a smartphone and a ranging device—both were held by a human participant. The participants were moving within the test area randomly. The test area was open to the public; however, there was light traffic during the experiments. The different smartphones used in the experiments were equipped with different sets of sensors. When inertial sensors were available, they were used to provide a PDR solution using the standalone filter. The rest of this section will evaluate the positioning performance of the main node, which was connected to the Pozyx system to collect the ground-truth trajectory.

The complete experiment consists of three separate trajectories. Each trajectory starts with the four nodes at rest. Once the experiment starts, the nodes move in random trajectories inside the testing area. The nodes occasionally stop on one of the reference position markers on the floor. The reference trajectory of the main node is captured using the Pozyx reference system. The data logging application runs on each of the smartphones and collects readings from the available sensors, the Wi-Fi received signal strength indicator along with information about the corresponding access points, and the UWB range measurement along with the address of the collaborating node.

The raw data of the three trajectories are processed using different scenarios. The first scenario evaluates the standalone positioning performance of the main node. In this scenario, all the sensors available to the main node are used by the positioning filter. The Wi-Fi fingerprint map used in this scenario is the reference map, which was created previously using a dedicated run. The objective of this scenario is to establish a performance baseline to which the performance of the collaborative approach is compared.

The next two scenarios are collaborative positioning scenarios. In the first collaborative scenario, the main node is not using any of the available sensors, except for the UWB ranging device. At the same time, the supporting nodes are estimating their positions using all the sensors available to them, along with the Wi-Fi reference map. The main node uses only the relative range measurements to estimate its position. The objective of this scenario is to evaluate the effect of collaboration in the case of node asymmetry. The main node, in this case, is in a disadvantageous position where it could not estimate its location without external aid from the collaborating nodes.

In the second collaborative scenario, the main node and the supporting nodes use the complete set of available sensors. The objective of this test is to assess the effect of collaboration when the active node already has a good estimate of its position using only measurements local to the device, without any external sources.

In the final collaborative scenario, the positioning filter is providing position estimates based on a random-walk model only. This final scenario, though seems trivial, is used to establish the lower bound of the positioning performance.

The rest of this section is divided into three subsections. Section 4.2.1 describes the process of generating the ground-truth trajectory of each of the test trajectories. It will also elaborate on the process of generating the Wi-Fi RSSI fingerprint maps. Section 4.2.2 is dedicated to the standalone performance using all the sensors available to the dynamic node, and without using any collaboration or relative range measurements for the positioning. Finally, Section 4.2.3 discusses the performance of the collaborative positioning approach. In this subsection, different collaboration scenarios are evaluated.

#### 4.2.1. Reference Trajectories and Fingerprints Maps

**Figure 14:**
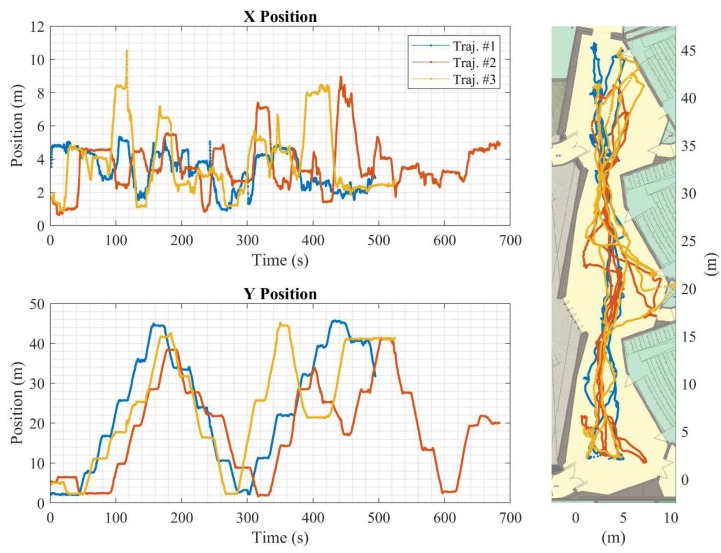
Pozyx reference trajectory.

The positioning performance of the LocSpeck system is evaluated using three different trajectories covering the same test area. Figure 14 shows the reference solution for the test trajectories. This reference is created using the Pozyx UWB-based system. The locations of the Pozyx anchors are highlighted in Figure 13. The position error is evaluated at the pre-surveyed reference points.

The reference solution for each trajectory is compared to the pre-surveyed reference points on the ground. The performance of the Pozyx solution is summarized in Table 7. For all the tested scenarios, the position error is evaluated when the main node reaches and stops over one of the reference points. This event is captured from the Pozyx reference trajectory in addition to the stop detection algorithm applied to the accelerometer data from the node of interest. The Pozyx trajectory is not used directly to evaluate the performance. It is used to indicate the location of the nearest reference point on the floor, which location is known precisely, and this reference point is used to evaluate the error in the position estimate. The small positioning error of the Pozyx system is vital to be able to distinguish between the densely placed reference points.

The reference radio map is created using the Pozyx reference trajectory in a separate run. The fingerprint map is built by observing the signal strength indicator at the reference points, then fit a Gaussian process model for each visible access point, using the position and signal strength pairs. During the positioning scenarios, the resulting Gaussian process models are used by the different dynamic nodes to aid the positioning filter.

#### 4.2.2. Standalone Positioning Results

The standalone scenario results comprise three trajectories for the main node. Due to the stochastic nature of the particle filter, each trajectory is run through the positioning filter 20 times to produce more robust statistics of the filter performance. The standalone positioning error statistics for the three trajectories are summarized in Table 8. For this scenario, the main node is using all the sensors available onboard the smartphone, i.e., gyroscope, accelerometer, and Wi-Fi information. The root-mean-square (RMS) positioning error across the three trajectories ranges from 4.28 to 6.65 m, while the overall RMS positioning error, in this case, is 5.92 m, as shown in Table 8. Since these results depend mainly on Wi-Fi fingerprinting, the performance might be affected by the presence of high human mobility in the test area [56]. Additionally, the overall performance of the standalone positioning scenario can be improved by augmenting the solution with other techniques, such as the geomagnetic field anomalies or visual scene recognition [57,58,59,60]. However, the main objective of the standalone filter in this work is to form the performance baseline, to which the effect of collaboration between nodes is to be measured, as discussed later in Section 4.2.3.

Table 9 shows the results of the IPIN competition winners from 2015 to 2018 [61,62]. These results are shown for comparison with the achievable performance of the standalone mode of the LocSpeck framework. The 75% percentile of the position error is not far from the top indoor positioning system available, although the winner of the 2018 off-site track can achieve 1.1 m accuracy.

It is worth noting that the inclusion of the results in Table 9 does not imply that the different systems can be compared directly since the performance of any positioning system will vary according to the operating conditions. The sole purpose of showing these results is to give a sense of the performance of the current state-of-the-art systems. The performance of the standalone solution acts as a baseline to which the collaborative positioning approach is evaluated.

#### 4.2.3. Collaborative Positioning Results

In this section, two collaboration scenarios are considered. The first scenario consists of four nodes, the main node, and three supporting nodes. The main node will not use any of its onboard sensors. However, the main node will only use the UWB device to measure the relative ranges between itself and the other collaborating nodes. The other supporting nodes will use all the sensors available to them, along with the range measurement device. The objective of this scenario is to evaluate the achievable performance using relative range measurements to dynamic nodes. The second scenario is similar to the first one with one change: the main node will be using all the available sensors, in addition to the range measurement device. The objective of this scenario is to assess the effect of the collaboration on the participating nodes. In addition to these two cases, the results of the positioning using the random-walk model only is showed as well.

• *Positioning using relative range measurements*

Table 10 shows the performance summary for the collaborative positioning approach, using the relative range measurement only. As expected, the performance, in this case, is worse than the performance of the standalone case. However, in this scenario, the mobile node is using only the range measurements, without any of the onboard sensors. In this case, the use of the collaborative positioning framework improves the positioning error for the main node by 50%, above the performance of the random-walk model only.

**Figure 15:**
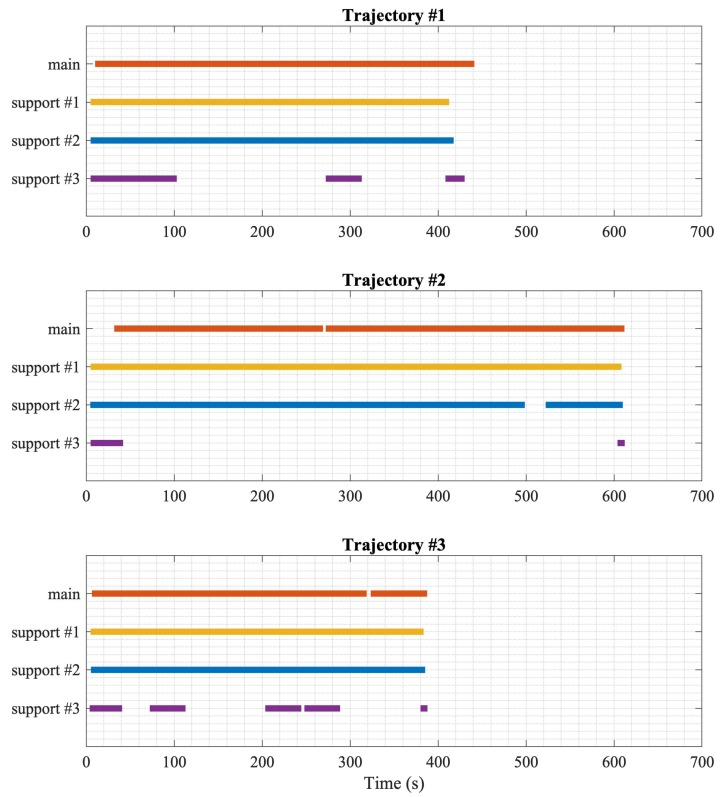
Nodes activity summary.

Another factor that can affect the performance of the main node in the collaborative setting is the availability of the supporting nodes. The availability of the nodes is illustrated in Figure 15, where each horizontal line represents the activity of the corresponding node. The gaps in the lines indicate that the node is not active. Although there are three supporting nodes, only two of them are active most of the time, and the third is fluctuating between the active and inactive states. The effect of the node availability is evident in the second trajectory, which has the most significant errors among the three trajectories.

• *Positioning using relative range measurements and all sensors*

This scenario evaluates the effect of the collaboration on the main node while using the full set of sensors available on board. When using all sensors, the main node should achieve a performance level similar to the performance of the standalone solution. Table 11 shows a summary of the positioning performance of the collaborative positioning, while the main node is using all its sensors. The collaboration negatively affected the performance of the main node, when it uses all the sensors. The mean error has increased by 27%, the RMS error by 16.6%, and the 75% percentile error by 32.2%.

• *Positioning using random-walk model only*

Before proceeding to evaluate the performance of the collaborative approach using relative range measurements, it would be useful to consider the error in the absence of the collaboration between the main node and the other nodes. Table 12 shows the positioning error statistics in this case. Without collaboration, the mean of the position error is 18.46 m, while the RMS of the position error is 21.60 m. The 75% percentile of the error is 26.57 m. The objective of this scenario is to establish a lower bound on the positioning performance.

## 5. Discussion

**Figure 16:**
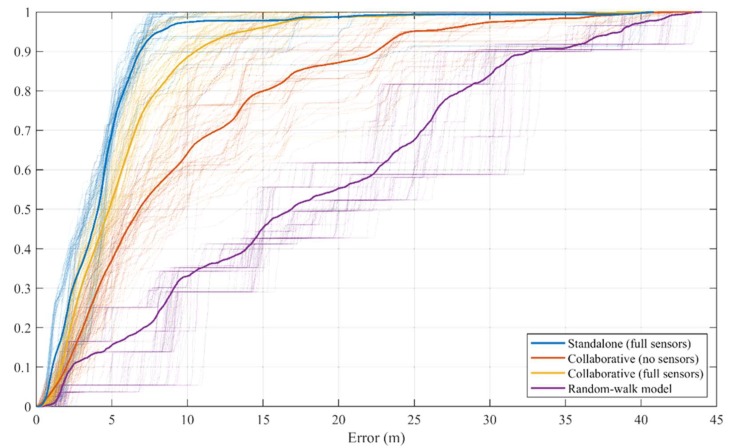
The combined positioning error CDF for the four scenarios.

This previous section provided an overview of the positioning performance using the LocSpeck collaborative framework. Figure 16 shows the cumulative distribution function (CDF) of the position error of the main node in the four scenarios described earlier. Table 12 lists the overall performance for each of the tested scenarios. It is evident that using the collaborating framework provides a significant advantage to nodes with little or no sensors. Without fusing information from the collaborating nodes, these weak nodes will not be able to estimate their position.

However, for the strong nodes in the process, the performance can suffer a hit. One possible explanation of the performance degradation could be related to the fusion of an erroneous estimate from one of the collaborating nodes. This case is exacerbated when the filter of a collaborating node suffers from particle depletion. As a result, the filter will generate new particles through resampling. These particles will have less diversity, and their corresponding covariance will be small. When a filter with these characteristics collaborates with another, it will provide false confidence in its position estimate. Consequently, it will drive the estimates of the collaborating nodes in the wrong direction. The performance degradation due to an erroneous state estimate or an overconfident remote note could be mitigated by implementing filter integrity measures to ensure that each filter has a realistic covariance estimate, possibly by utilizing the actual values of the measurement likelihood function. Such measures could include divergence monitoring [63] or by increasing the number of particles to better resembles the posterior of the filter.

## 6. Conclusions

This paper described and evaluated the LocSpeck system, a collaborative and multimodal framework for indoor positioning using smartphones. The collaboration between the different nodes is achieved using a dynamic and ad-hoc network. The network architecture and a light-weight medium access control protocol were highlighted and evaluated. The paper provided an overview of the hardware and software components of the individual nodes.

Different simulations and experiments were performed to evaluate the performance of the standalone positioning algorithm of the nodes, along with the performance of the collaborative positioning approach. These experiments confirm the advantage of the proposed framework, as discussed. The results shown in this paper demonstrate that using collaborative positioning approaches can provide pronounceable improvement in the performance, especially for situations involving asymmetric nodes, where the weak nodes can benefit from the superior sensing or capabilities available in nearby nodes. However, the performance of the system can be improved by implementing state integrity checks to ensure that the positioning filters will not suffer from depletion, which can negatively affect the overall performance of the system.

## Figures and Tables

**Table 1:** The LocSpeck BLE GATT profile.

Characteristic	Properties	Size (bit)	Description
DEVID	R	32	DW1000 Device ID
PARTID	R	32	DW1000 Lot ID
OTPREV	R	8	DW1000 OTP Revision
Range	R (Notify)	32	Range measured
Pair ID	R (Notify)	16	The ID of the paired node
CONF	R/W	16	Node settings
Node ID	R	16	Node ID

**Table 2:** Node settings.

Node Setting	Value
Channel number	5
Pulse repetition frequency (PRF)	64 MHz
Preamble length (PLEN)	1024
Data rate (DR)	110 kbps
Range between nodes	60 cm

**Table 3:** Ranging messages size and duration.

Messages	Size (byte)	Duration (ms)
Blink	12	2.57
Initiate	22	3.32
Poll	12	2.57
Response	16	2.87
Final	20	3.18
Report	16	2.87
**Total**		17.38

**Table 4:** Range value and frame duration statistics.

	Range Error (cm)	Frame Duration (ms)
Mean	1.6	43.1
Standard Deviation	3.5	2.0

**Table 5:** Theoretical maximum ranging rate.

**Frame Duration**		43.10 ms
**Guard Interval**	+	15%
**Total Frame Duration**	=	49.57 ms
**Ranging Frame Rate**		20.17 Hz

**Table 6:** Ranging rate maxima.

	Channel	Node
Number of Nodes	18	5
Ranging Rate	7.9	2.1

**Table 7:** Pozyx positioning error summary.

Trajectory #	Mean Absolute Error (m)	RMS Error (m)
1	0.37	0.45
2	0.57	0.65
3	0.60	0.72

**Table 8:** Standalone positioning results summary.

Error Stats. (m)	Traj. #1	Traj. #2	Traj. #3	Overall
Mean	3.80	4.33	4.84	4.36
Min	0.27	0.02	0.28	0.02
Max	9.14	40.84	21.85	40.84
50% Percentile	3.34	3.77	4.41	4.09
75% Percentile	5.30	5.62	5.14	5.34
90% Percentile	6.82	7.13	6.57	6.85
RMS	4.28	6.65	5.75	5.92
Std. dev.	1.97	5.04	3.10	4.01

**Table 9:** Performance of indoor positioning competitions (75% percentile).

Competition	Track	Accuracy (m)
IPIN 2015	Smartphone (on-site)	6.6
IPIN 2015	Smartphone (off-site)	8.3
IPIN 2016	Smartphone (on-site)	5.4
IPIN 2016	Smartphone (off-site)	5.8
IPIN 2017	Smartphone (on-site)	8.8
IPIN 2017	Smartphone (off-site)	3.48
IPIN 2018	Non-Camera based Positioning (on-site)	5.5
IPIN 2018	Smartphone (off-site)	1.1

**Table 10:** Collaborative positioning results summary (no sensors).

Error Stats. (m)	Traj. #1	Traj. #2	Traj. #3	Overall
Mean	8.58	10.65	8.36	9.51
Min	0.02	0.00	0.06	0.00
Max	34.61	43.21	27.44	43.21
50% Percentile	6.49	6.99	6.70	6.79
75% Percentile	10.91	16.48	12.89	13.38
90% Percentile	15.90	23.80	16.77	22.42
RMS	10.57	14.32	10.15	12.43
Std. dev.	6.18	9.58	5.77	7.99

**Table 11:** Collaborative positioning results summary (all sensors).

Error Stats. (m)	Traj. #1	Traj. #2	Traj. #3	Overall
Mean	5.98	5.36	5.49	5.54
Min	0.03	0.01	0.01	0.01
Max	27.26	42.35	23.08	42.35
50% Percentile	4.44	4.96	4.84	4.81
75% Percentile	8.97	6.94	6.35	7.06
90% Percentile	11.16	10.00	9.02	10.36
RMS	7.30	6.92	6.54	6.90
Std. dev.	4.18	4.37	3.55	4.11

**Table 12:** Positioning Results Summary.

Error Stats. (m)	Standalone (Full Sensors)	Collaborative (Full Sensors)	Collaborative (No Sensors)	Random-Walk
Mean	4.36	5.54	9.51	18.46
Min	0.02	0.01	0.00	0.25
Max	40.84	42.35	43.21	43.79
50% Percentile	4.09	4.81	6.79	17.89
75% Percentile	5.34	7.06	13.38	26.57
90% Percentile	6.85	10.36	22.42	32.63
RMS	5.92	6.90	12.43	21.60
Std. dev.	4.01	4.11	7.99	11.2
